# Terpenoids and Bibenzyls from Three Argentine Liverworts

**DOI:** 10.3390/molecules161210471

**Published:** 2011-12-16

**Authors:** Fumihiro Nagashima, Yoshinori Asakawa

**Affiliations:** Faculty of Pharmaceutical Sciences, Tokushima Bunri University, Yamashiro-cho, Tokushima 770-8514, Japan; Email: asakawa@ph.bunri-u.ac.jp (Y.A.)

**Keywords:** Jungermanniales, liverwort, *Radula voluta*, *Frullania brasiliensis*, *Anastrophyllum* species, bibenzyl, rosane, chemosystematics

## Abstract

A new rosane diterpenoid, 3α-hydroxy-5,15-rosadien-11-one (**3**), was isolated, together with a known rosane diterpenoid, 5,15-rosadiene-3,11-dione (**4**), and an aromadendrane sesquiterpenoid, *ent*-cyclocolorenone (**5**), from the Et_2_O extract of an unidentified Argentine liverwort *Anastrophyllum *species. Moreover, four known sesquiterpene lactones **6**–**9** and two known bibenzyls **10**, **11** were isolated from the Et_2_O extracts of Argentine *Frullania brasiliensis *and *Radula voluta*, respectively. The structures of compounds **3**–**11** were determined by the use of NMR techniques.

## 1. Introduction

**Figure 1:**
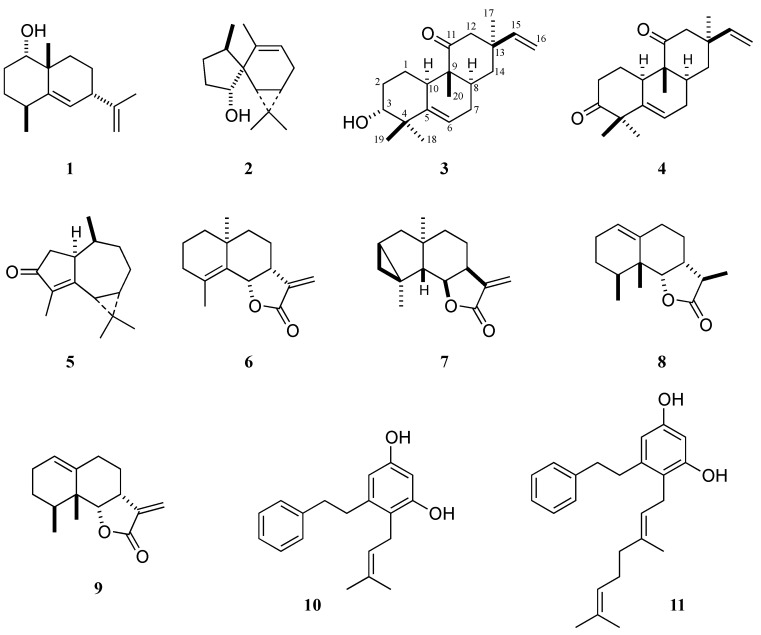
Terpenoids and bibenzyls isolated from Argentine liverworts.

Due to their small morphology, liverworts (Hepaticae) are difficult to classify and identify. However, they are a rich source of terpenoids and aromatic compounds, which can be used to evaluate their chemosystematics [1,2]. In our search for new biologically active substances, we continue to study the chemical constituents of liverworts. Many liverworts are endemic to the southern hemisphere, including Oceania and South America. Recently, we have reported the structures of new sesqui- and diterpenoids from New Zealand liverworts [3]. We also reported the isolation of new sesquiterpenoids **1** and **2** from an unidentified *Gackstroemia* species from New Zealand [4]. During the course of our investigation of the chemical constituents of three Argentine Jungermanniales species (unidentified *Anastrophyllum* species, *Radula voluta* and *Frullania brasiliensis*), we isolated a new rosane diterpenoid **3** and six previously known compounds: Rosane **4**, aromadendrane **5**, two eudesmanes **6** and **7**, two eremophilanes **8** and **9**, and two bibenzyls **10** and **11** (Figure 1) and characterized their chemical structures. The chemosystematics of these three species from Argentina are discussed.

## 2. Results and Discussion

The new rosane diterpenoid **3** was isolated from the ether extract of an unidentified *Anastrophyllum* species by chromatographic separation (silica gel and Sephadex LH-20), together with *ent*-cyclocolorenone (**5**) [5] and 5,15-rosadiene-3,11-dione (**4**) [6], whose spectral and physical data were identical with those of authentic samples.

The mass spectrum of **3** showed *m/z* 302 [M]^+^ and its molecular formula, C_20_H_30_O_2_ (calcd. 302.2245), was confirmed by HR-EIMS. The IR spectrum demonstrated the presence of hydroxy and carbonyl groups. The ^1^H-NMR spectrum (Table 1) of **3** showed the signals of terminal vinyl protons (δ 4.94 *dd*, 4.96 *dd*, 5.83 *dd*), an olefinic proton (δ 5.56 *d*), a proton (δ 3.23 *dd*) on a carbon bearing a hydroxy group and four tertiary methyls. The ^13^C-NMR spectrum (Table 2) exhibited 20 carbons, and its DEPT spectrum indicated the presence of trisubstituted olefinic carbons (δ 117.0 *d*, 145.5 *s*), terminal vinyl carbons (δ 110.2 *t*, 147.9 *d*), a carbonyl carbon (δ 214.7) and a methine (δ 77.2) with a hydroxy group, together with four methyls, four methylenes, two methines and three quaternary carbons. Since the ^13^C-NMR (Table 2) data was similar to those of the 5,15-rosadiene-3,11-dione (**4**) [6], compound **3 **was suggested to be a rosane diterpenoid.

**Table 1:** ^1^H-NMR data of **3** (600 MHz, CDCl_3_) ^a^.

H		H	
1	1.01 m	14	1.36 dd (10.2, 2.5) α
	1.97 m		1.76 m β
2	1.69 m	15	5.83 dd (17.3, 10.7)
	1.78 m		
3	3.23 dd (11.5, 4.7)^b^	16	4.94 dd (10.7, 0.8)
			4.96 dd (17.3, 0.8)
6	5.56 d (6.3)	17	0.97 s
7	1.87 m	18	0.99 s
	1.98 m		
8	1.77 m	19	1.15 s
10	2.72 m	20	0.98 s
12	1.95 dd (12.6, 2.5) α		
	2.73 d (12.6) β		

^a^ Chemical shift values are in δ (ppm); ^b^ Coupling constants are in Hz.

**Table 2:** ^13^C-NMR of **3** and **4** (100 MHz, CDCl_3_) ^a^.

C	3	4 [6]	C	3	4 [6]
1	25.3	25.2	11	214.7	214.4
2	30.3	37.8	12	48.4	48.5
3	77.2	214.6	13	41.6	41.6
4	42.1	51.0	14	38.4	38.4
5	145.5	144.2	15	147.9	147.7
6	117.0	118.2	16	110.2	110.4
7	28.9	29.0	17	23.5	23.5
8	38.3	38.2	18	21.4	22.7
9	48.9	49.4	19	24.4	29.5
10	37.9	38.6	20	12.7	12.0

^a^ Chemical shift values are in δ (ppm).

**Figure 2:**
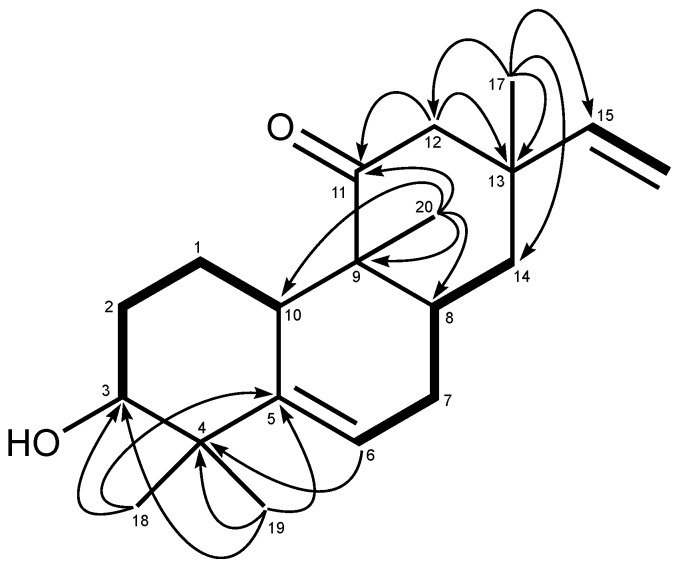
^1^H-^1^H (bold line) and long range ^1^H-^13^C (arrows) correlations of **3**.

**Figure 3:**
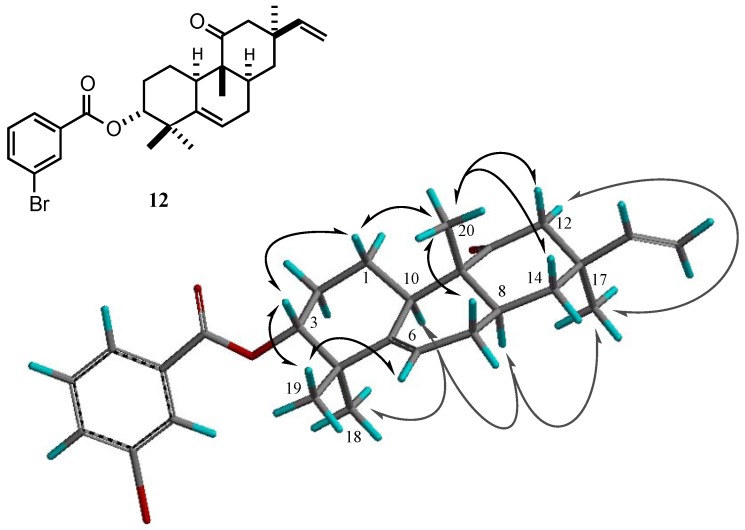
NOE correlations of **12**.

The ^1^H-^1^H COSY of **3** confirmed three partial segments: (A) -CH(OH)-CH_2_-CH_2_-CH-, (B) -CH_2_-CH-CH_2_-CH=C-, and (C) –CH=CH_2_. As seen in the HMBC spectrum (Figure 2), the tertiary methyl at Η−17 correlated with the methylene carbon at C-4 in segment B, the terminal vinyl carbon at C-15, the quaternary carbon at C-13 and the isolated methylene carbon at C-12, methylene protons at H-12 of which correlated with the carbonyl carbon at C-11. The other tertiary methyl at H-20 correlated with the methine at C-10 in segment A, the carbonyl carbon, the quaternary carbon at C-9 and the aliphatic methine in segment B. The methyl groups at H-18 and 19 correlated with the methine at C-3 bearing hydroxy group in segment A, an aliphatic quaternary carbon and a trisubstituted olefinic quaternary carbon. On the basis of the above results, the structure of **3** was elucidated to be 3-hydroxy-5,15-rosadien-11-one. The stereochemistry of **3** was clarified by the NOESY spectrum of the *m*-bromobenzoate derivative **12** of **3**. NOE correlations (Figure 3) of **12** were observed between: (i) H-3 and H-1β, H-18; (ii) H-1β and H-20; (iii) H-20 and H-7β, H-12β, H-14β; (iv) H-19 and H-10; (v) H-10 and H-8; (vi) H-8 and H-17. Moreover the CD spectrum of **3** showed a positive Cotton effect (λ_max_ 297) as the same Cotton effect (λ_max_ 298) as **4** [6]. Thus, the structure of **3** was shown to be 3α-hydroxy-5,15-rosadien-11-one. However, the absolute configuration of **3** has not yet clarified because the use of only the Cotton effect of the CD spectrum was not able to establish it unequivocally.

*Frullania brasiliensis* contains sesquiterpene lactones [7]. We reexamined the same species for the purpose of potentially isolating new compounds. A chromatographic separation of the ether extract of *Frullania brasiliensis* resulted in the isolation of two eudesmanes, (+)-frullanolide (**6**), which causes potent allergenic contact dermatitis [8], and nepalensolide A (**7**) [9], and two eremophilanes, 5-*epi*-dilatanolide A (**8**) [7] and 5-*epi*-dilatanolide B (**9**) [7].

Two bibenzyl compounds, 3,5-dihydroxy-2-(3-methyl-2-butenyl)bibenzyl (**10**) [10,11] and 2-geranyl-3,5-dihydroxybibenzyl (**11**) [12], were chromatographically isolated from the ether extract of *Radula voluta*.

Fusicoccane and sphenolobane diterpenoids are common chemical components isolated from *Anastrophyllum* species, e.g., from *A. minutum* [13], *A. aurztum* [14] and *A. donnianum* [15]. These diterpenoids are very characteristic for *Anastrophyllum* genus belonging to the Jungermanniaceae. However, neither sphenolobane nor fusicoccane were isolated from the present unidentified *Anastrophyllum* species. Those isolates included naturally rare rosane diterpenoid and aromadendrane sesquiterpenoid. Therefore, the unidentified *Anastrophyllum* species may be from a chemically different taxon from the other *Anastrophyllum* species.

Eudesmane and eremophilane sesquiterpene lactones have been isolated from *F. brasiliensis* (Frullaniaceae) [7] and are considered the most important chemical markers of the Frullaniaceae [1,2,16]. The present *F. brasiliensis* also contained the same eudesmanolides and eremophilanolides as those reported.

A number of bibenzyls and prenyl bibenzyls were isolated from European, New Zealand, Ecuador and Japanese *Radula* species (Radulaceae) [1,2,17]. *R. voluta* also produced bibenzyls **10** and **11**, which are ubiquitous components in the *Radula* species. The *Radula* including *R. voluta *is chemically very isolated from the other liverworts examined so far, since the presence of terpenoids is very rare.

## 3. Experimental

### 3.1. General

^1^H and ^13^C-NMR: 200, 400 and 600 MHz (^1^-NMR) and 100, 150 MHz (^13^C-NMR). Chemical shift values were expressed in δ (ppm) downfield from tetramethylsilane as an internal standard (^1^H-NMR) and δ 77.03 (ppm) from CDCl_3_ as a standard (^13^C-NMR). TLC: Visualized under UV (254 nm) light and by spraying with 10% H_2_SO_4_ or Godin reagent [18] followed by heating at 120–130 °C. MeOH-CH_2_Cl_2_ (1:1) was used for Sephadex LH-20. [α]_D_: CHCl_3_. *Radula voluta *Tayl. ex Gott., Lindenb. & Nees, *Frullania brasiliensis* Raddi and unidentified *Anastrophyllum* species were collected in Argentina in 2005 and identified by Prof. Dr. S. R. Gradstein (University of Göttingen, Germany). The voucher specimen was deposited at the Institute of Pharmacognosy, Tokushima Bunri University.

### 3.2. Extraction and Isolation

The dry material (14.7 g) of the unidentified *Anastrophyllum* species was ground and extracted with Et_2_O. The crude extract (471.4 mg) was divided into 10 fractions by column chromatography on silica gel (*n*-hexane-EtOAc gradient). Fraction 4 gave *ent*-cyclocolorenone (**5**) ([α]_D_ +376.9° *c* 1.45; 15.6 mg) and 5,15-rosadiene-3,11-dione (**4**) (6 mg) by rechromatography on silica gel (*n*-hexane-EtOAc 17:3, CH_2_Cl_2_-Et_2_O 49:1). 3α-Hydroxy-5,15-rosadien-11-one (**3**) (4.5 mg) was purified from Fraction 6 by Sephadex LH-20 and silica gel (*n*-hexane-EtOAc 19:1).

The crude Et_2_O extract (1.9 g) of *F. brasiliensis *(90.8 g) was chromatographed on silica gel (*n*-hexane-EtOAc gradient) to give six fractions. Fraction 3 was rechromatographed on Sephadex LH-20, silica gel (*n*-hexane-EtOAc or *n*-hexane-Et_2_O), MPLC (Si-60, toluene) and prep. HPLC (Cosmosil 5SL-II, *n*-hexane-Et_2_O 98:2) to give (+)-frullanolide (**6**) (59.3 mg) and nepalensolide A (**7**) (15.1 mg). 5-*Epi*-dilatanolide A (**8**) (13.9 mg) and 5-*epi*-dilatanolide B (**9**) (5.4 mg) were isolated from Fraction 5 by a combination of reverse phase silica gel (CH_3_CN), MPLC (Si-60, *n*-hexane-Et_2_O 4:1) and prep. HPLC (UK-silica, *n*-hexane-EtOAc 9:1).

The dry material (690 mg) of *R. voluta* was ground and extracted with Et_2_O. The crude extract (79 mg) was chromatographed on Sephadex LH-20 and silica gel to give 3,5-dihydroxy-2-(3-methyl-2-butenyl)bibenzyl (**10**) (17.6 mg) and 2-geranyl-3,5-dihydroxybibenzyl (**11**) (3.1 mg).

*3α-Hydroxy-5,15-rosadien-11-one* (**3**): [α]_D_ +71.3° (*c* 1.31); CD (EtOH): Δε_297nm_ +1.70, Δε_211nm_ −0.51 (*c *= 9.77 × 10^−4^); FTIR ν_max_ cm^−1^: 3438, 1704; ^1^H-NMR see Table 1; and ^13^C-NMR see Table 2; HR-EIMS: calcd for C_20_H_30_O_2_: 302.2245. Found: 302.2253; EIMS *m/z* (rel. int.): 302[M]^+^(10), 284(100), 269(47), 241(76), 226(11), 211(11), 201(11), 187(47), 173(47), 171(46), 159(23), 151(34), 145(21), 134(27), 119(26), 105(29), 91(25), 79(113), 67(11), 55(13), 41(15).

*m-Bromobenzoate* **12**:To compound **3 **(3.5 mg) in dry CH_2_Cl_2_ (2 mL) was added *m*-bromobenzoic acid (5 mg), DCC (4 mg) and DMAP (2 mg) and the solution was stirred at r.t. overnight. The reaction mixture was filtered and chromatographed on silica gel (*n*-hexane-EtOAc 19:1) to yield *m*-bromobenzoate **12** (4.2 mg). ^1^H-NMR (600 MHz, CDCl_3_): δ 8.17 (1H, *t*, *J *= 1.9 Hz), 7.98 (1H, *dd*, *J *= 8.0, 1.1 Hz), 7.69 (1H, *ddd*, *J *= 8.0, 1.9, 1.1 Hz), 7.33 (1H, *t*, *J *= 8.0 Hz), 2.05 (1H, *m*, H-1α), 1.16 (1H, *dd*, *J *= 12.9, 4.1 Hz, H-1β), 1.82-1.95 (3H, *m*, H-2, H-2, H-7α), 4.73 (1H, *dd*, *J *= 11.3, 4.7 Hz, H-3), 5.62 (1H, *d*, *J *= 6.3 Hz, H-6), 1.99 (1H, *m*, H-7β), 1.80 (1H, *m*, H-8), 2.81 (1H, *brd*, *J *= 12.4 Hz, H-10), 1.97 (1H, *dd*, *J *= 12.6, 2.2 Hz, H-12α), 2.74 (1H, *d*, *J *= 12.6 Hz, H-12β), 1.38 (1H, *dd*, *J *= 9.9, 2.5 Hz, H-8α), 1.77 (1H, *d*, *J *= 12.4 Hz, H-14β), 5.84 (1H, *dd*, *J *= 17.3, 10.7 Hz, H-15), 4.95 (1H, *dd*, *J *= 10.7, 0.8 Hz, H-16), 4.97 (1H, *dd*, *J *= 17.3, 0.8 Hz, H-16), 0.99 (3H, *s*, H-17), 1.22 (3H, *s*, H-18), 1.10 (3H, *s*, H-19), 1.02 (3H, *s*, H-20); ^13^C-NMR (100 MHz, CDCl_3_): δ 214.6, 164.8, 147.9, 144.5, 135.8, 132.8, 132.6, 130.0, 128.2, 122.5, 117.9, 110.2, 80.0, 49.0, 48.4, 41.6, 41.2, 38.4, 38.3, 37.9, 28.9, 26.7, 25.0, 24.6, 23.5, 23.1, 12.6.

## 4. Conclusions

A new rosane diterpenoid **3** was isolated from the unidentified Argentine liverwort *Anastrophyllum *species, together with a known rosanediterpenoid **4 **and an aromadendranesesquiterpenoid **5**. The known bibenzyls **10** and **11** and sesquiterpene lactones **6**–**9** were isolated from Argentine *Radula voluta *and *Frullania brasiliensis*.

## References

[1] Asakawa Y., Herz W., Grisebach H., Kirby G.W. (1982). Chemical constituents of the Hepaticae. Progress in the Chemistry of Organic Natural Products.

[2] Asakawa Y., Herz W., Kirby G.W., Moore R.E., Steglich W., Tamm Ch. (1995). Chemical constituents of the Bryophytes. Progress in the Chemistry of Organic Natural Products.

[3] Asakawa Y., Toyota M., Nagashima F., Hashimoto T. (2008). Chemical constituents of selected Japanese and New Zealand liverworts. Nat. Prod. Commun..

[4] Nagashima F., Kuba Y., Ogata A., Asakawa Y. (2010). Sesqui- and diterpenoids from three New Zealand liverworts *Bazzania novae-zelandiae*, *Gackstroemia* sp*. *and *Dendromastigophora *sp. Nat. Prod. Res..

[5] Matsuo A., Nakayama M., Sato S., Nakamoto T., Uto S., Hayashi S. (1974). (−)-Maalioxide and (+)-cyclocolorenone, enantiomeric sesquiterpenoids from the liverwort, *Plagiochila*
*acanthophylla* subsp. japonica. Experientia.

[6] Feld H., Zapp J., Becker H. (2003). Secondary metabolites from the liverwort *Tylimanthus renifolius*. Phytochemistry.

[7] Bardón A., Mitre G.B., Kamiya N., Toyota M., Asakawa Y. (2002). Eremophilanolides and other constituents from the Argentine liverwort *Frullania brasiliensis*. Phytochemistry.

[8] Asakawa Y., Muller J.-C., Ourisson G., Foussereau J., Ducombs G. Nouvelles lactones sesquiterpéniques de *Frullania* (Hépaticae). Isolement, structures, propriétés allergisantes. Bull. Soc. Chim. France.

[9] Tori M., Miyazaki N., Kondo K., Taira Z., Asakawa Y. (1990). Nepalensolide A, Novel sesquiterpene lactone from the liverwort *Frullania nepalensis*. Compound breaking the Samek rule. A study by NOE and X-ray. Chem. Lett..

[10] Asakawa Y., Toyota M., Takemoto T. (1978). Seven bibenzyls and a dihydrochalcone from *Radula variabilis*. Phytochemistry.

[11] Crombie L.W., Crombie W.M.L., Firth D.F. (1988). Synthesis of bibenzyl cannabinoids, hybrids of two biogenetic series found in *Cannabis sativa*. J. Chem. Soc. Perkin Trans. 1.

[12] Asakawa Y., Kondo K., Tori M., Hashimoto T., Ogawa S. (1991). Prenyl bibenzyls from the liverwort *Radula*
*kojana*. Phytochemistry.

[13] Beyer J., Becker H., Toyota M., Asakawa Y. (1987). Diterpenoids with a novel skeleton from the liverwort *Anastrophyllum minutum*. Phytochemistry.

[14] Zapp J., Burkhardt G., Becker H. (1994). Sphenolobane and fusicoccane diterpenoids from the liverwort *Anastrophyllum*
*aurztum*. Phytochemistry.

[15] Buchanan M.S., Connolly J.D., Rycroft D.S. (1996). Sphenolobane diterpenoids from the liverwort *Anastrophyllum*
*donnianum*. Phytochemistry.

[16] Asakawa Y. (2004). Chemosystematics of Hepaticae. Phytochemistry.

[17] Kraut L., Mues R., Zinsmeister H.D. (1997). Prenylated bibenzyl derivatives from *Lethocolea*
*glossophylla* and *Radula voluta*. Phytochemistry.

[18] Godin P. (1954). A new spray reagent for paper chromatography of polyols and cetoses. *Nature* (*London*).

